# Incidence, clinical predictors, and clinical effect of new-onset atrial fibrillation in myocardial infarction patients: A retrospective cohort study

**DOI:** 10.15537/smj.2022.43.8.20220349

**Published:** 2022-08

**Authors:** Fayez Elshaer, Abdulelah H. Alsaeed, Sultan N. Alfehaid, Abdulaziz S. Alshahrani, Abdulrahman H. Alduhayyim, Ayman M. Alsaleh

**Affiliations:** *From the Department of Cardiac Sciences (Elshaer, Alsaleh); from the College of Medicine (Elshaer, Alsaeed, Alfehaid, Alshahrani, Alduhayyim), King Saud University Medical City, King Saud University, Riyadh, Kingdom of Saudi Arabia; and from the Department of Cardiology (Elshaer), National Heart Institute, Cairo, Egypt.*

**Keywords:** atrial fibrillation, myocardial infarction, predictors

## Abstract

**Objectives:**

To calculate the incidence of new-onset atrial fibrillation (NOAF) in myocardial infarction (MI) patients and examine associated predictors and clinical outcomes of NOAF patients.

**Methods:**

A retrospective cohort study was used to carry out this study. All MI patients admitted to King Khaled University Hospital, Riyadh, Saudi Arabia, between January 2015 to 2020 were eligible for inclusion. The study excluded those with a previous diagnosis of atrial fibrillation and patients who died at presentation.

**Results:**

A total of 281 patients were analyzed with a mean age of 58.7±12.7. Incidence of NOAF was 7.8%. Significant predictors identified by multivariate logistic regression analysis included older age (*p*=0.004), history of MI (*p*=0.012), and undergoing coronary artery bypass graft surgery (CABG) as treatment (*p*=0.016). New-onset atrial fibrillation was associated with higher odds of major adverse cardiovascular event (*p*=0.039), ventricular tachycardia (*p*=0.001), and mortality (*p*=0.031).

**Conclusion:**

New-onset atrial fibrillation is a relatively common complication of MI, and in our study, it was associated with higher odds of further complications including death. Therefore, identification of MI patients at risk of developing NOAF is crucial. Our study suggests that older age, a previous history of MI, and undergoing CABG are significant predictors of NOAF development.

**N**ew-onset atrial fibrillation (NOAF) is a common complication of myocardial infarction (MI). Reported incidence rates ranged between 6% and 21%.^1^ Development of NOAF in an MI patient is associated with other complications and worse outcomes including heart failure, stroke, and cardiogenic shock.^2^ Moreover, NOAF patients have a higher odds of mortality with an odds ratio (OR) of 1.37 according to a systematic review.^2,3^ As a result, determining NOAF predictors can assist with early detection and potentially improve outcomes. Known factors that increase the risk of developing NOAF after MI include older age, congestive heart failure, hypertension, and high heart rate at admission.^1,2,4-9^ In regard to the predictors of NOAF, older age is one of the most consistent predictors reported regardless of the study design or region.^4,5,7,8^ However, not all predictors are as well established as older age. For example, the use of gender as a predictor of NOAF produced opposite results in different studies. Some studies found that male gender was a predictor of NOAF, while other studies found that gender was not a predictor when multivariate regression analysis was carried out.^4,5,7^ Instead, they found female gender to be associated with the development of NOAF.^4,7^ This shows the importance of further studies to investigate previously studied predictors in order to provide more data regarding the topic.

Recent studies have found new associated factors. A 2017 study found that cardiogenic shock is a predictor of NOAF.^4^ Another example is a study by Parashar et al^5^ which reported a significant association between NOAF and high levels of C-reactive protein and N-terminal-pro-B-type natriuretic peptide (BNP). Further studies are needed to investigate these factors in populations that have not been previously studied, such as people in Saudi Arabia or other Arab nations. This study aimed to calculate the incidence of NOAF and examine associated predictors and clinical outcomes in a cohort of patients from Saudi Arabia with MI.

## Methods

The study was carried out as a retrospective cohort study from September 2020 to December 2021. Before making the study design, we carried out a review of the existing literature. A search of MEDLINE and Google scholar was carried out with the MeSH terms “atrial fibrillation” and “myocardial infraction”. We then filtered out the results to include studies carried out regarding NOAF only.

**Figure 1:**
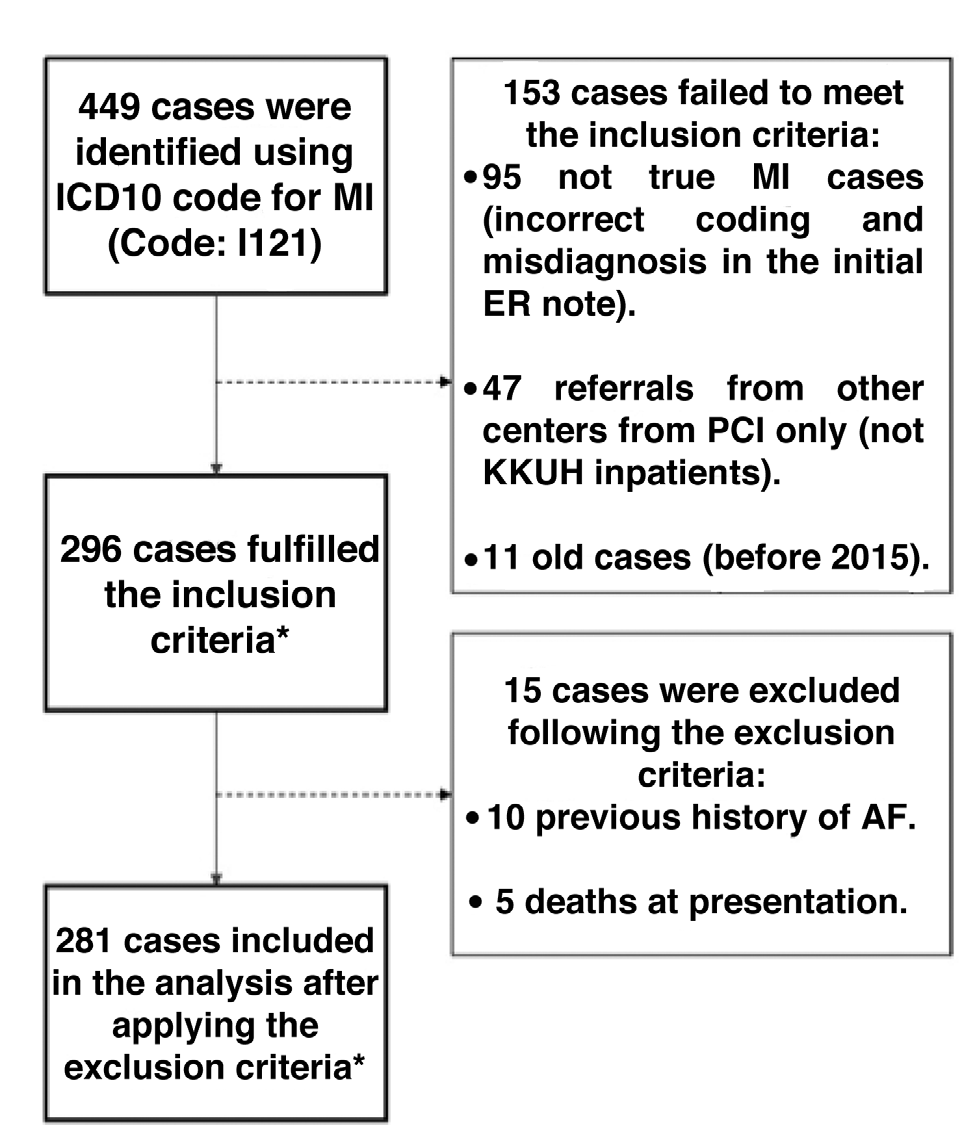
- Data collection pathway. *Inclusion criteria: I) King Khaled University Hospital (KKUH) patients who were admitted for primary treatment of MI as inpatients; II) admission between January 2015 to January 2020. **Exclusion criteria: I) patients who had a confirmed pervious diagnosis of AF before the development of MI; II) patients who died at presentation. ICD: International Classification of Diseases, MI: myocardial infarction. ER: Emergency room, PCI: percutaneous coronary intervention, AF: atrial fibrillation

Patients who were admitted to King Khaled University Hospital (KKUH) (a tertiary care hospital), Riyadh, Saudi Arabia, with a diagnosis of MI between January 2015 and 2020 were eligible for inclusion. Data were collected from the medical records. Patients were identified using the International Classification of Diseases-10 codes for MI (I121). We then removed the cases that failed to meet the inclusion criteria (incorrect coding, old cases, and MI patients transferred only for percutaneous coronary intervention [PCI] from other hospitals). We excluded patients who had a confirmed pervious diagnosis of atrial fibrillation before the development of MI and patients who died at presentation. Patients were retrospectively followed up for one year. A study flow chart of the data collection pathway is shown in Figure 1.

The variables examined in the study included demographics (age, gender, body mass index [BMI], and smoking status), comorbidities and past medical history (prior MI, hypertension, diabetes, dyslipidemia, congestive heart failure, structural heart disease, chronic lung disease, asthma, mental disorders, dementia, and any other illnesses). Moreover, the study also investigated the baseline clinical admission data (heart rate, systolic blood pressure, diastolic blood pressure, type of MI, Killip class, and presentation timing). Late presentation was defined as >6 hours between MI symptom onset and hospital arrival. Biomarker and electrolyte levels at presentation were also investigated (C-reactive protein, BNP, troponin I, creatinine, sodium, potassium, and urea). This was detected using the lab results of the blood sample obtained on arrival. Details regarding the management were investigated including medications on arrival and during admission, type of treatment (PCI, coronary artery bypass graft [CABG], and medical therapy). Lastly, the heart rate and systolic and diastolic blood pressure were recorded at the 6- and 12-month follow-ups. The outcome variable was development of NOAF. Post MI complications were also recorded for up to 12 months.

The study was approved by the King Saud University Institutional Review Board (Research ID: E-20-5141). All of the steps carried out in this study were in accordance with the Helsinki Declaration.

### Statistical analysis

Statistical analyses were carried out using the Statistical Package for the Social Sciences, version 25.0 (IBM Corp., Armonk, NY, USA). The study had a subgroup sample for patients who completed a full 6- and 12- months follow-up. The data were first analyzed using Chi-square (for categorical data) and independent t-test (for the quantitative data) for the analysis of the baseline categorical and quantitative clinical and demographic characteristics. This analysis included the following variables: demographics, baseline clinical admission data, biomarkers, electrolytes at presentation, and follow-up data. Quantitative variables were expressed as means with standard deviation (SD), while categorical variables were expressed as numbers or frequencies with percentage. Afterwards, the study carried out a multivariant logistic regression analysis of the patients’ clinical characteristics to identify the independent predictors. This analysis included variables from all the previous categories. The variables chosen for this analysis were the variables that were significant in the Chi-square analysis, and the ones that were significant in previous literature. Lastly, the study then ran a backward stepwise elimination algorithm on the comorbidities of the patients, complications developed after the MI and the medications given on arrival and during treatment. This was carried out to reduce the variables until only the significant predictors (*p*<0.05) remained in the multivariate logistic regression. Missing values in this analysis were addressed using multiple imputation. The missing values were the following: 5 patients with missing BMI during admission; 3 missing location and type of MI; 2 missing presenting heart rate and troponin; and one patient missing systolic and diastolic blood pressure. The major missing variable was BNP, with 32 patients. This is most likely because it is not part of the routine investigations of acute coronary syndrome in the emergency room in KKUH, Riyadh, Saudi Arabia. A *p*-value of <0.05 and 95% confidence interval (CI) were used to report the statistical significance and precision of results in all steps of the analysis.

The STROBE cohort checklist was used to ensure proper reporting of the study.^10^

## Results

A total of 296 MI patients were eligible for study inclusion. Ten patients were excluded because of a history of atrial fibrillation and 5 for death at presentation. Finally, 281 patients were included for analysis. Among these, 119 patients completed 6- and 12-month follow-up visits (follow-up subgroup cohort). Overall, mean age was 58.7±12.7 years, and 232 (82.6%) patients were men. Prevalence of comorbidities was as follows: diabetes, 176 patients (62.6%); hypertension, 160 (56.9%); obesity (BMI >30 kg/m^2^), 87 (31.0%), and smoking, 106 (37.7%). The incidence of NOAF was 22 (95% CI: [5.2-11.7]). Mean age in patients who developed NOAF was 66.6±12.0 years and 19 (86.4%) were men.

Table 1 shows baseline categorical clinical and demographic data in patients grouped according to development of NOAF. Older age category (age >55) patients had a statistically significant higher proportion of NOAF (*p*=0.01). Also, a significantly higher proportion of patients in the NOAF group died (*p*=0.031).

**Table 1:** - Categorical baseline clinical and demographic characteristics.

Variables	Development of NOAF					95% CI	
	n (%)	Yes	Total	*P*-value	OR	Lower	Upper
***Age at visit (binned)***							
56+	146 (56.4)	19 (86.4)	165 (58.7)	0.01	5.756	1.507	21.99
***Gender***							
Male	213 (82.2)	19 (86.4)	232 (82.6)	0.45	1.687	0.431	6.601
***Body mass index***							
>30	80 (30.9)	7 (31.8)	87 (31.0)	0.46	0.684	0.251	1.867
***Smoking status***							
Smoker	98 (37.8)	8 (36.4)	106 (37.7)	0.75	1.169	0.444	3.079
***Hypertension***							
Yes	144 (55.6)	16 (72.7)	160 (56.9)	0.218	1.983	0.667	5.89
***Diabetes***							
Yes	163 (62.9)	13 (59.1)	176 (62.6)	0.059	0.346	0.115	1.042
***Type of MI***							
STEMI	163 (62.9)	10 (45.5)	173 (61.6)	0.23	0.575	0.231	1.430
NSTEMI	96 (37.1)	12 (54.5)	108 (38.4)				
***Presentation timing***							
Late	57 (22.0)	5 (22.7)	62 (22.1)	0.72	1.220	0.410	3.635
***Presenting heart rate***							
>100	45 (17.4)	3 (13.6)	48 (17.1)	0.78	1.208	0.320	4.563
***Follow-up outcome and complications***							
Developed complications	104 (40.2)	11 (50.0)	115 (40.9)	0.40	0.673	0.267	1.695
***Mortality***							
Died during hospitalizing or follow-up	17 (6.6)	5 (22.7)	22 (7.8)	0.031	4.629	1.152	18.593

Values are presented as number and percentage (%). OR: odds ratio, CI: confidence interval, NOAF: new-onset atrial fibrillation, MI: myocardial infarction, STEMI: ST elevation myocardial infarction, NSTEMI: non-ST elevation myocardial infarction

Table 2 shows the quantitative baseline clinical and demographic data in patients grouped according to development of NOAF for both the entire cohort and the follow-up cohort. In the entire cohort, age was significantly older in the NOAF group (*p*=0.002); in addition, both BNP (*p*=0.046) and urea (*p*=0.047) levels at presentation were found to be significantly associated with NOAF. In the follow-up cohort, none of the variables significantly differed between the NOAF group and non-NOAF group.

**Table 2:** - Quantitative baseline clinical and demographic characteristics.

Variables	Development of NOAF			95% CI	
	No	Yes	*P*-value	Lower	Upper
***Full sample***					
Age at visit	58.1±13.0	66.6±12.0	0.002	-13.98	-3.07
BMI	27.9±4.6	27.6±4.4	0.769	-1.70	2.30
Presenting heart rate	84.6±19.0	86.1±20.0	0.715	-10.08	6.92
Presenting systolic blood pressure	137.1±25.0	138.1±25.0	0.846	-11.96	9.81
Presenting diastolic blood pressure	79.1±15.0	80.3±18.0	0.728	-7.84	5.48
B-type natriuretic peptide	2497.0±4122.0	6286.3±8333.8	0.046	-7514.07	-64.57
Troponin I	9686.9±15788.6	8288.0±15494.3	0.690	-5495.60	8293.58
Creatinine	105.8±81.9	172.3±215.2	0.164	-162.42	29.34
Sodium	135.5±11.3	135.9±3.6	0.875	-5.16	4.40
Potassium	4.3±0.6	4.4±0.7	0.481	-0.38	0.18
Urea	6.5±4.4	10.1±7.8	0.047	-7.00	-0.05
***Follow-up sample***					
Heart rate at 6 months follow-up	72.0±13.0	73.0±14.0	0.926	-7.64	6.95
Systolic blood pressure at 6 months follow-up	130.0±21.0	127.0±21.0	0.669	-9.63	14.95
Diastolic blood pressure at 6 months follow-up	72.0±12.0	67.0±12.0	0.217	-2.55	11.14
Heart rate at 12 months follow-up	72.0±15.0	76.0±17.0	0.445	-12.80	5.65
Systolic blood pressure at 12 months follow-up	130.0±21.0	130.0±16.0	0.964	-12.93	12.36
Diastolic blood pressure at 12 months follow-up	72.0±13.0	69.0±14.0	0.431	-4.75	11.06

Values are presented as mean ± standard deviation (SD). OR: odds ratio, CI: confidence interval, NOAF: new-onset atrial fibrillation, BMI: body mass index

Multivariate regression analyses were carried out for the entire cohort, with the results shown in Table 3. Older age was found to be a significant predictor of the development of NOAF (OR=1.088, 95% CI: [1.028-1.152]; *p*=0.004). Compared to patients who underwent PCI, the odds of NOAF were almost 8 times higher in those who underwent CABG (OR=7.950; 95% CI: [1.473-42.897]; *p*=0.016). Patients who developed a major adverse cardiovascular event (MACE) during PCI had 12 times higher odds of developing NOAF (OR=12.15; 95% CI: [1.133-130.220]; *p*=0.039). Multivariate logistic regression analyses were then carried out in the follow-up cohort, which is shown in Table 4, and it included follow-up variables. In this cohort, older age was significantly associated with development of NOAF (OR=1.083; 95% CI: [1.009-1.164]; *p*=0.028).

**Table 3:** - Logistic regression analysis for demographics, baseline clinical admission data, biomarkers, electrolytes at presentation.

Variables	OR	95% CI		*P*-value
		Lower	Upper	
Age at visit	1.088	1.028	1.152	0.004
Gender (male)	1.458	0.289	7.365	0.648
Smoking status (smoker)	2.331	0.658	8.255	0.190
Type of MI (STEMI)	0.433	0.119	1.573	0.204
Presentation (late)	1.666	0.446	6.216	0.448
Killip class (overall Killip class effect with Class 4 as the reference category)				0.454
Killip class 1	5.733	0.304	108.138	0.244
Killip class 2	7.407	0.398	137.746	0.179
Killip class 3	1.576	0.043	57.894	0.805
Presenting heart rate	1.005	0.976	1.034	0.742
Presenting systolic blood pressure	0.978	0.950	1.007	0.136
Presenting diastolic blood pressure	1.039	0.995	1.085	0.087
Brain natriuretic peptide	1.000	1.000	1.000	0.137
Troponin I	1.000	1.000	1.000	0.748
Creatinine	1.000	0.994	1.007	0.974
Sodium	1.011	0.957	1.068	0.694
Potassium	1.064	0.456	2.480	0.886
Urea	1.023	0.895	1.169	0.744
Treatment given (overall treatment given effect with PCI as the reference category)				0.039
CABG	7.950	1.473	42.897	0.016
Medical treatment	0.753	0.116	4.891	0.767
Major adverse cardiac event (MACE) during PCI	12.145	1.133	130.220	0.039

OR: odds ratio, CI: confidence interval, NOAF: new-onset atrial fibrillation, MI: myocardial infarction, STEMI: ST elevation myocardial infarction, CABG: coronary artery bypass graft surgery

**Table 4:** - Logistic regression analysis for demographics, baseline clinical admission data, biomarkers, electrolytes at presentation and follow-up data.

Variables	OR	95% CI		*P*-value
		Lower	Upper	
Age at visit	1.083	1.009	1.164	0.028
Gender (male)	1.165	0.208	6.527	0.862
Smoking status (smoker)	1.984	0.209	18.808	0.551
Type of MI (STEMI)	0.432	0.101	1.852	0.258
Presenting heart rate	1.006	0.966	1.047	0.787
Presenting systolic blood pressure	0.975	0.937	1.013	0.192
Presenting diastolic blood pressure	1.035	0.974	1.101	0.267
NT-pro-brain natriuretic peptide	1.000	1.000	1.000	0.968
Troponin I	1.000	1.000	1.000	0.456
Creatinine	0.989	0.965	1.014	0.392
Sodium	1.020	0.915	1.137	0.726
Potassium	0.910	0.315	2.631	0.862
Urea	1.069	0.807	1.417	0.640
Heart rate at 6 months follow-up	0.998	0.932	1.069	0.956
Systolic blood pressure at 6 months follow-up	0.989	0.938	1.043	0.685
Diastolic blood pressure at 6 months follow-up	0.990	0.907	1.080	0.817
Heart rate at 12 months follow-up	1.013	0.956	1.074	0.654
Systolic blood pressure at 12 months follow-up	1.014	0.968	1.062	0.562
Diastolic blood pressure at 12 months follow-up	0.989	0.920	1.062	0.751

OR: odds ratio, CI: confidence interval, NOAF: new-onset atrial fibrillation, MI: myocardial infarction, STEMI: ST elevation myocardial infarction

Backward stepwise elimination was then carried out using patient comorbidities, complications, and medications administered (Table 5). This was carried out to reduce the variables until only the significant predictors remained in the multivariate logistic regression (*p*<0.05). The overall model was statistically significant (χ^2^=27.713, *p*<0.05). Patients who had a history of prior MI were associated with a higher OR of developing NOAF. New-onset atrial fibrillation patients were associated with higher odds of developing ventricular tachycardia after the MI and were more likely to receive unfractionated heparin and low molecular weight heparin. Moreover, they had lower odds of receiving clopidogrel, ticagrelor or an oral hypoglycaemic agent.

**Table 5:** - Logistic regression analysis using backward stepwise elimination for comorbidities, complications, and medications administered.

Variables	OR	95% CI		*P*-value
		Lower	Upper	
Prior MI	4.040	1.359	12.011	0.012
Clopidogrel	0.332	0.117	0.942	0.038
Oral hypoglycaemic	0.266	0.078	0.913	0.035
Unfractionated heparin	5.014	1.606	15.659	0.006
Ticagrelor	0.297	0.096	0.919	0.035
Low molecular weight heparin	3.268	1.101	9.707	0.033
Ventricular tachycardia	12.297	2.941	51.418	0.001
Constant	0.057			0.000

The overall model was statistically significant (χ^2^=27.713, *p*<0.05). OR: odds ratio, CI: confidence interval, MI: myocardial infarction

## Discussion

In this study, NOAF developed in 7.8% of MI patients, which was similar to the incidence reported in previous studies.^1^ Older age, certain biochemical markers, previous history of MI, type of treatment, and medication received were significant predictors of NOAF. Patients with NOAF had higher odds of developing MACE, ventricular tachycardia, and a higher mortality.

Older age has been reported as a significant predictor of NOAF in almost all previous NOAF studies.^1,2,4-8^ Our study found similar results. Degenerative age-related cardiac changes such as fibrosis are established risk factors for atrial fibrilation.^11^ Moreover, older age is associated with increased risk of MI complications; therefore, higher incidence of NOAF would be expected in older patients.^12^ Our study also found that history of MI was significantly associated with development of NOAF. Similar to age, MI is associated with degenerative cardiac changes such as fibrosis and an increased risk of complications from any subsequent MI.^13^ Furthermore, patients with multiple MIs are more likely to be older and have multiple comorbidities, which puts them at higher risk for NOAF.^13^

We also investigated levels of biochemical markers, including troponin I, BNP, creatinine, urea, and electrolytes. However, only BNP and urea levels significantly differed between the NOAF and non-NOAF groups. N-terminal-pro-B-type natriuretic peptide is released by the ventricles because of muscular stretching. Elevated BNP level in the acute setting is an important marker of congestive heart failure.^14^ Previous studies have reported that elevated BNP is an independent predictor of NOAF, which was confirmed in a recent large prospective study.^5,15,16^ While studies investigating the association of NOAF after MI and urea level are very limited, our study found that urea level significantly differed between MI patients who did and did not develop NOAF. However, a similar finding has been reported in coronavirus disease 2019 patients.^17^ Urea is elevated in patients with decreased glomerular filtration rate (suggestive of kidney failure), congestive heart failure, and dehydration.^18^ In our study, BNP (a marker of heart failure) was also significantly higher in patients who developed NOAF; therefore, urea and BNP may have been elevated together in patients with heart failure.

Type of treatment and medications received also significantly differed between patients who developed NOAF and those who did not. The odds of NOAF were much higher in MI patients who underwent CABG compared to those who underwent PCI. This was consistent with what was reported by a previous study based on the Global Registry of Acute Coronary Events.^19^ This association might be due to the fact that patients who underwent CABG instead of PCI are generally older and have more comorbidities and more advanced cardiovascular disease, which are characteristics associated with NOAF development.^19,20^ New-onset atrial fibrillation patients were more likely to receive unfractionated heparin and low molecular weight heparin and less likely to receive clopidogrel and ticagrelor. This finding was consistent with those of other studies and was likely due to the concern of increased risk of bleeding.^7,19^

In our study, odds of mortality were higher in patients who developed NOAF. A previous systematic review reported that NOAF was associated with a higher risk of mortality in MI patients even after adjusting for confounders.^3^ We also found that MI patients with NOAF were more likely to experience a MACE after PCI than those who did not develop NOAF. Moreover, they were more likely to develop ventricular tachycardia. The association between NOAF and the development of ventricular tachycardia was also found by other studies.^7,19^ However, the number of patients in our study who experienced a MACE or ventricular tachycardia were limited. Further studies are warranted to confirm our findings.

### Study limitations

Because of its retrospective and observational nature, cause and effect cannot be demonstrated. The study was carried out in a single center, so the sample size was limited. Furthermore, our findings may not necessarily be generalizable to patients with characteristics that differ from our study population, such as patients with pre-existing atrial fibrillation or NOAF patients without MI.

In conclusion, the incidence of NOAF in MI patients was 7.8%. Older age, history of MI, and undergoing CABG were predictors of NOAF in multivariate analysis. New-onset atrial fibrillation patients were more likely to experience a MACE or ventricular tachycardia and had a higher mortality. This study provided data regarding NOAF patients in an Arabic population which had limited data regarding the topic. The study’s finding of the association between the high levels of urea and the development of NOAF was a novel finding in the context of NOAF post-MI. The study also confirms the dangers of NOAF and how it leads to higher rates of mortality and complications. This shows the importance of the early detection and treatment of NOAF. Further large-scale prospective studies are needed, especially in the Arabic region, to confirm our findings. Also, clinical trials would be better suited at investigating the role of medications in NOAF development.
